# Motor Responses and Weight Gaining in Neonates through Use of Two Methods of Earmuff and Receiving Silence in NICU

**DOI:** 10.1155/2014/864780

**Published:** 2014-12-30

**Authors:** Z. Abdeyazdan, S. Ghasemi, M. Marofi, N. Berjis

**Affiliations:** ^1^Nursing and Midwifery Care Research Center, Faculty of Nursing and Midwifery, Isfahan University of Sciences, Isfahan, Iran; ^2^Student Research Center Committee, Faculty of Nursing and Midwifery, Isfahan University of Sciences, Isfahan, Iran; ^3^Pediatric Department, Faculty of Nursing and Midwifery, Isfahan University of Sciences, Isfahan, Iran; ^4^ENT Department, Medical School, Isfahan University of Sciences, Isfahan, Iran

## Abstract

*Background and Aims*. With technological advances in NICUs the survival rate of preterm infants has been increased. Because NICU environment is a potent source of stress for infants, its modification is an essential measure to decrease infants' morbidity. The purposes of this study were to compare the effects of wearing earmuff and provision silence for infants on their motor responses and gaining weight. *Methods*. In a randomized clinical trial 96 preterm infants were enrolled. Their motor responses were evaluated for two consecutive days in the morning and afternoon shifts, in the groups of earmuff and silence, and at similar time points in the control group. Also their weight was measured at days 1 and 10. *Results*. In the two intervention groups, means of motor responses in infants were significantly less than in the control group, and weight gain of infants was more than the control group. However weight gain was more pronounced in the earmuff group. *Conclusion*. Both interventions led to decreasing number of motor responses and improvement of weight gain pattern, but these effects were more pronounced in earmuff group; thus because implementation of silence in NICUs has many barriers, it is suggested to use earmuff for preterm infants in these units. This trial obtained IRCT registration number IRCT2012092010812N2.

## 1. Introduction

Auditory development starts from 23 to 24 weeks of gestational age. At this time fetal auditory threshold is approximately 65 dB. Auditory system development continues during intrauterine life, and the threshold is gradually diminished to that of an adult level [1]. In addition, some parts of neonatal auditory system develop shortly after birth [2]. Existence of appropriate sensory stimulants is essential for normal growth and development. In fact, environment influences fetal and neonatal development through various senses such as visual, auditory, tactile, olfactory, and taste sensation [3]. An imbalance between sensory stimuli and brain development stage leads to an injury in the neonates. In fact, neonates' surrounding environment should be balanced with their stage of development [4]. Intrauterine environment provides the fetus with ideal conditions for its growth and development, and amniotic fluid and uterine wall act as a protector for the fetus. During intrauterine life fetus is exposed to several auditory stimulants originating from internal and extraneous interference. Most of these sounds have a specific pattern and rhythm. Extrauterine sound reaches the fetus after being modified by intrauterine wall [4], which protects fetal auditory system [5]. Over 70% of premature neonates need hospitalization in NICU, where they are exposed to numerous auditory stimulants for which they are not developed enough [6]. Noise pollution is accompanied by increasing the risk of hearing loss in infants [7]; in addition it can act as a stressor leading to an increase in heart rate, metabolism, and energy needs as well as a reduction in the energy storage, needed for neonatal growth and development [8]. Stress results in secretion of hormones that contribute to more fat and protein catabolism in the body. Thus, the neonates, tolerating stress, may have slower weight gaining leading to prolonged time to be discharged from NICU [9]. In recent years, several studies have been conducted to evaluate neonatal responses to noise reduction. These studies are categorized in two major groups: those on the reduction of noise production from existing sources in NICU [10, 11] and those on prevention of receiving noise by neonates [12, 13]. Although the efficiency of environmental noise reduction interventions to diminish the stress imposed to neonates has been reported in various studies, and attention to environmental noise, as one of the influencing factors on the neonates, is a part of nursing care [14], these methods are not given as a constant strategy in NICUs in many countries including Iran. It seems that provision of some quiet hours in such a professional, innovative, and sophisticated ward is a difficult task. Therefore, suggesting a constant applicable strategy is essential. To the best of our knowledge, no study concerning the effect of various intervention methods for reduction of neonates' exposure to acoustic stimulants has been already taken. The present study aimed to compare the effect of earmuff usage and silence provision on premature infants' motor responses and their weight gaining.

## 2. Materials and Methods

In a clinical trial, 96 premature infants meeting inclusion criteria were assigned to three groups (group one: subjects with earmuffs, group two: control, and group three: subjects receiving silence). Research environment was NICU of Shahid Beheshti Hospital affiliated to Isfahan University of Medical Sciences, Iran. The premature infants met inclusion criteria including gestational age of 29–36 weeks, APGAR ≥ 7 in the first and the fifth minute after birth, no brain problems, major congenital anomalies, and sepsis, no need for mechanical ventilation, and normal hearing. Sudden physiologic instability in infants and parents' will to withdraw from the research were the exclusion criteria.

### 2.1. Sampling

Since the longevity of effect of staff education to perform silence in NICU environment was unpredictable, wearing earmuffs was considered as the first intervention. The subjects were selected through convenient sampling to be assigned to either group one or group two (wearing earmuffs or control) and then were put in each of study and control groups by tossing a coin. Finally, the sampling for the third group (group of silence) was randomly done among the hospitalized premature infants who met inclusion criteria.

### 2.2. Procedure

After obtaining the ethical code from the Ethics Committee of Isfahan University of Medical Sciences, one of the researchers introduced herself to the hospital manager and NICU head nurse explained the study's purpose and methods and attained their permission for doing research.

**Figure 1:**
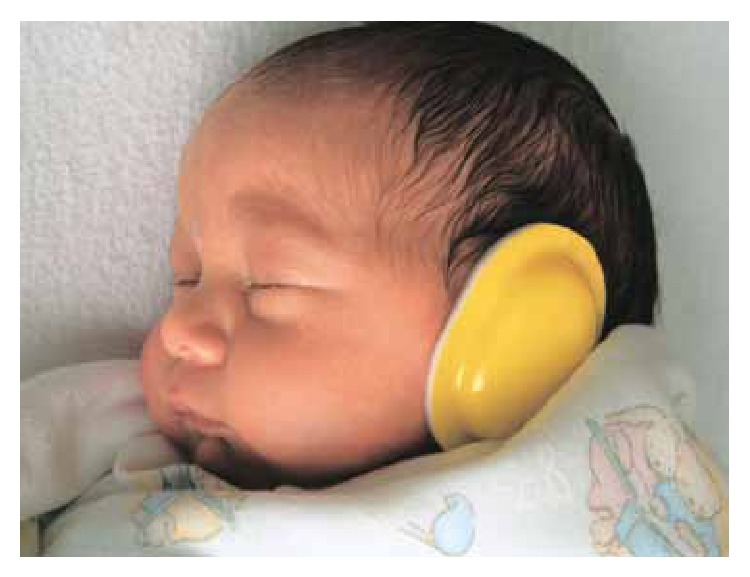
Newborn puts on earmuff.

Sampling was conducted after obtaining an informed written consent from the infants' parents. Before sampling, the staff received necessary education in relation to both methods of intervention (usage of earmuffs and the designed method for silence provision) and highly emphasized that they had no limitation for administration of treatment procedures and nursing care for the infants during the interventions. Earmuffs were put on the external ear of the infants (Figure 1). Silence intervention protocol included both behavior modification and NICU environment modification. Both interventions (wearing earmuffs and silence program) were conducted during the busiest time of morning (9–11 am) and evening (4–6 pm) shifts for duration of two hours in two consecutive days and during the night (11 pm−5 am) for ten straight nights. Motor responses including tremor, twitch, and startle reflex were evaluated by observation and their frequency was calculated in periods of 15 minutes, before, during, immediately after, and one hour after interventions in the mornings and evenings in both groups of intervention and on identical time points in control group. Infants' weight was measured in identical hours in all three groups in the morning, with the same conditions (before feeding, with clean napkin, and naked). In order to be sure about the reduction of sound intensity during the intervention of silence as well as imposing no difference between two groups of control and earmuffs, sound intensity was measured nearby infants with an identical distance from their heads by a sound level meter. Standard sound level meters and scales were used to measure needed variables.

### 2.3. Reliability and Validity

Infants' weight was measured before and during 10 days of intervention by the researcher with an identical scale (a one-kilo control weight was used for scales reliability). Environmental noise was measured by a digital sound level meter 641105 made by Vogel Germany GmbH & Co. KG.

Earmuffs used for infants were approved by the Council of Europe and American National Standard Institute (ANSI), decreased received sound intensity at least by 7 dB, and led to reduction of sound pressure level (SPL) >50%.

Accuracy of digital sound level meter was arrested with other existing devices in this sphere. In addition, the device was tested in an equipped laboratory and calibrated through a comparative method. Seca medical scales, made in France with accuracy of ±10 gr, were adopted.

### 2.4. Data Analysis

The data were analyzed by descriptive and inferential statistical tests (mean, SD, paired *t*-test, repeated measure ANOVA and one-way ANOVA, and LSD post hoc) through SPSS18.

## 3. Results

In this study, 108 infants entered the study, of whom 12 were excluded: four in earmuffs group (three due to discharge and one due to apnea), five in silence group (due to unload before the conclusion of the intervention), and three in the control group (due to discharge before the end of study).

Results showed no significant difference in demographic characteristics between three groups (Table 1).

Mean numbers of motor responses during, immediately after, and one hour after the intervention showed a significant difference between three groups. Also mean numbers of motor responses in each group of earmuffs, silence, and control showed a significant difference in various time points (Table 2). LSD post hoc test showed that mean numbers of motor responses have significant differences in the morning shifts during, immediately after, and also one hour after intervention between earmuffs and silence groups, earmuffs and control group, and also between silence and control groups. In the evening shifts, the mean differences of motor responses were significant during and immediately after intervention between earmuffs and each of silence and control groups and between silence and control groups, at one hour after the interventions, between earmuffs and silence, and also between silence and control groups (Table 3).

With regard to mean differences of motor responses in each group in various time points, LSD post hoc showed a significant difference between times before and after intervention both in the morning and in the afternoon, before and immediately after the intervention, and before and one hour after intervention in earmuff group. In silence group, there was a significant difference just at times before and during and before and one hour after intervention. In the control group, the difference was only significant for similar time points before and during intervention (Table 4).

With regard to infants' weight gaining, the findings showed that average weights at the first day and at the end of the intervention were 1489 (460.5) and 1573 (493.7) g, respectively, in the first group (earmuff). In other words, this group had average of 83.7 gr weight gain during this period.

In the second group (control), mean weights at the first day and tenth day were 1616.3 (489.1) and 1624.24 (462.1) g, respectively. Put differently, these infants gained an average of 7.94 gr weight during 10 days.

In the third group (silence), mean weights at the first day and at the end of the intervention were 1524.2 (567) and 1583.3 (571.7) g, respectively. In other words, this group had an average of 59.1 gr weight gain during 10 days. Paired *t*-test showed no significant difference between the mean of infants' weights in the first and the last days of intervention in each group of earmuff, silence, and control. One-way ANOVA showed a significant difference in mean of weight increase in three groups (*P* = 0.005). LSD post hoc showed no significant difference in mean weight increase between earmuff and silence group (*P* = 0.3), but the difference between earmuff and control (*P* = 0.002) and silence and control (*P* = 0.01) groups was significant.

## 4. Discussion

Results showed a reduction in behavioral responses during use of earmuffs, which is consistent with study of Duran et al. [15]. These responses increased after removal of earmuffs, but they remained less than those before intervention for one hour after intervention. This constant effect has also been reported in studies of Trapanotto et al. [16] and Zahr and de Traversay [17].

Implementation of a silence period could diminish the infants' motor responses, but this effect was not constant, and the responses increased just after intervention so that they showed no difference with those before intervention.

A former study, already conducted in Isfahan city, showed that, during the intervention of reduction of noise and light, the number of infants' motor responses diminished [11]. Slevin et al. in a study reduced the level of noise, light, and nonorganized infants' care by staff and showed that the mean number of infants' motor activities was brought down, compared to before intervention [10]. Although in the present study just noise was lowered, the obtained results are in line with two aforementioned studies. In control group, mean number of infants' motor responses increased through time, possibly due to increase of sound intensity in NICU. Previous researches investigated the effect of various interventions on infants' weight gain. Mann et al. demonstrated that reduction of sound and light led to increased infants' weight gain in intervention group compared to control [13]. Abou Turk et al. also showed that premature LBW and VLBW infants had more weight gain after wearing a silicon earmuff compared to control [12]. In the present study, the obtained results are in line with previous studies and support the fact that neonatal stress leads to energy expenditure, which may alter growth, so that infants exposed to stress have delayed discharge from the NICU [9]. Our obtained results showed no significant difference in the mean infants' weight increases between earmuff and silence groups during 10 days, but a significant weight increase was observed in both intervention groups compared to control group. Comparison of infants' weight at birth and at the end of intervention revealed an average of 44 g increase in earmuff group, while the infants in silence and control groups had weight reductions of 45 g and 42 g, respectively. In fact, infants' weight gain in earmuff group was better than in silence group, and in silence group it was better than in the control group.

## 5. Conclusion

To the best of our knowledge, the present study is the first to compare the effect of two intervention methods for noise stress reduction on infants' motor responses and their weight gaining pattern. The obtained results showed that both interventions led to infants' fewer motor responses and improvement of their weight gain trend, although this effect was more pronounced in earmuff group compared to silence group. Therefore, use of earmuffs can be suggested as one of the care strategies, particularly in the wards in which prevailing silence faces executive problems.

## Figures and Tables

**Table 1:** Demographic characteristics in three groups.

	Ear pad group		Silent group		Control group		^*^ *P* value
	Mean	SD	Mean	SD	Mean	SD	

Birth weight	1529.3	503.9	1631	558.9	1669.3	533.3	0.3
Gestational age (weeks)	31.4	2.8	31.9	1.7	31.8	2.6	0.7
Postnatal age (days)	7.1	4.2	5.9	2.5	5.03	3.5	0.2
APGAR score (first min)	7.4	0.7	8.1	0.7	7.5	0.8	0.5
APGAR score (fifth min)	8.5	0.8	8.9	1	8.6	0.9	0.6

^*^One-way ANOVA.

**Table 2:** Mean and SD of motor responses in various time points in three groups in the morning and afternoon.

	Motor responses	Earmuff group		Silent group		Control group		^*^ *P*
		Mean	SD	Mean	SD	Mean	SD	
Morning	BI	33	19.3	34.5	25.2	33.5	16.6	0.6
	DI	11.1	6.2	29.5	20.1	37	13.2	<0.000
	IAI	17.5	17.1	35.6	22.8	36.7	15	0.07
	OHAI	22.4	12.5	40.03	24.3	33.7	12.5	0.3
	^**^ *P*	0.02		0.000		0.000		

Afternoon	BI	29.3	18.6	27.3	14.6	25	10.8	0.6
	DI	9.8	7.4	25	10.8	25	10.8	<0.000
	IAI	17.6	3.6	22.6	15.8	22.8	15.8	0.05
	OHAI	19.9	15.7	23.03	13.3	23.3	13.3	0.3
	^**^ *P*			0.4		0.000		

^*^One-way ANOVA.

^**^Repeated measures ANOVA.

BI: before intervention, DI: during intervention, and IAI: immediately after intervention.

OHAI: one hr after intervention.

**Table 3:** Results of post hoc LSD test (P value) for comparison mean of infants' motor responses in paired groups.

	Groups	Time			
		Before	During	Immediately after	1 hr after
Morning	Earmuff & silence	0.2	0.000^*^	0.000^*^	0.000^*^
	Earmuff & control	0.6	0.000^*^	0.05^*^	0.000^*^
	Silence & control	0.2	0.03^*^	0.003^*^	0.000^*^

Afternoon	Earmuff & silence	0.6	0.02^*^	0.02^*^	0.000^*^
	Earmuff & control	0.6	0.000^*^	0.000^*^	0.4
	Silence & control	0.3	0.000^*^	0.01^*^	0.001^*^

^*^means that the difference is meaningful.

**Table 4:** Results of post hoc LSD test (*P* value) for comparison of mean of infants' motor responses in paired times in each group.

Time	Groups					
	Control		Silence		Earmuff	
	Morning	Afternoon	Morning	Afternoon	Morning	Afternoon
Before & during	0.06	0.05^*^	0.002^*^	0.3	0.000^*^	0.000^*^
Before & immediately after	0.3	0.1	0.5	0.1	0.001^*^	0.001^*^
Before & 1 hr after	0.7	0.001^*^	0.01^*^	0.2	0.006^*^	0.004^*^
During & immediately after	0.9	0.000^*^	0.001^*^	0.2	0.02^*^	0.000^*^
During & 1 hr after	0.01^*^	0.000^*^	0.000^*^	0.4	0.000^*^	0.000^*^
Immediately after & 1 hr after	0.1	0.03^*^	0.000^*^	0.9	0.01^*^	0.1

^*^means that the difference is meaningful.

## Limitations

In the present study, the researcher could not make major structural changes in the NICU environment for conducting silence due to economic and operational issues, so the silence program was only behavioral modification. As the aims of this study were not blind to the staff, this might have affected their care provision to infants as well as the researchers' evaluation of infants' motor responses, and these can be counted as limitations to the present study.
